# Modified subcostal arch xiphoid thoracoscopic expanded thymectomy for thymic carcinoma: a case report and review of literature

**DOI:** 10.1186/s13019-022-01981-w

**Published:** 2022-09-10

**Authors:** Jincheng Wang, Yang Liu, Wenmao Zhuang, Yinghao Zhao

**Affiliations:** 1 Department of Thoracic Surgery, The Second Hospital of Jilin University, Changchun, China; 2 People’s Hospital of Nongan County, Changchun City, Jilin Province China

**Keywords:** Thymic carcinoma, Thoracoscope, Modified subxiphoid subcostal arch enlarged thymectomy

## Abstract

Thymic neoplasms are a relatively uncommon tumor, with the anterior mediastinum being the most common. Median sternotomy is the procedure of choice for the treatment of thymomas. With the advent of thoracoscopy, an increasing number of countries are adopting the right thoracic approach for the treatment of thymomas, but there are still no clear surgical standards or modalities to treat thymic carcinoma. We propose a modified subxiphoid subcostal arch thoracoscopic enlarged thymectomy to treat thymic carcinoma based on various reviews. We have also reviewed the relevant literature on the subject of evidence-based medicine. The evaluation of CD70 in combination with CD5 and CD117 or preferentially expressed antigen in melanoma in combination with CD5 and CD117 may help to diagnose thymic squamous cell carcinoma (TSCC) more accurately. The modified thoracoscopic expanded thymic resection under the costal arch of the xiphoid process is not only suitable for TSCC but also for thymic cyst, thymoma, locally invasive thymoma, and thymic carcinoma.

## Introduction

The most common epithelial neoplasm in the anterior mediastinum is thymus epithelial neoplasm [1]. In 2015, World Health Organization (WHO) classified thymic epithelial neoplasms into types A, AB, B1, B2, B3, and C (thymic carcinoma, including thymic neuroendocrine carcinoma). The classification reflects tumors’ biological behavior and prognosis to a certain extent. According to the biological behavior differences of different tumor subtypes, tissue type was simplified into three subtypes: low-risk group (types A, AB, and B1), high-risk group (types B2 and B3), and thymic carcinoma group (type C). Subtypes of thymic carcinoma include squamous cell carcinoma, basal cell carcinoma, mucoepidermoid carcinoma, lymph epithelioma, clear cell carcinoma, sarcomatoid carcinoma, adenocarcinoma (papillary adenocarcinoma, has the characteristics of adenoid cystic carcinoma of the thymus gland carcinoma, mucous adenocarcinoma, and adenocarcinoma undifferentiated), the middle route of the testis nucleoprotein carcinoma, undifferentiated carcinoma, and other rare thymic tumors (gland scale cancer, liver cancer, and thymic carcinoma undetermined) [2]. Thymic carcinoma is a rare thymic epithelial malignant tumor with many histological subtypes. The histological subtypes of thymic carcinoma mainly include squamous and lymphoepithelioma-like carcinoma, which account for 60–70% of cases [3]. Thymic carcinoma often presents as an aggressive clinical presentation. Although there is no standard treatment for thymic carcinoma and surgical outcomes vary considerably between studies, a large series of operations based on a multi-agency or crowd-based database have proven the clinical benefit of surgery for thymic carcinoma [4]. Myasthenia gravis has little effect on the prognosis of thymoma, but it is suitable for early diagnosis and treatment. However, all patients with thymoma, regardless of myasthenia gravis, should be treated with enlarged thymic resection [5].

This study mainly analyzed a 63-year-old male patient who was found to have a thymic tumor during physical examination, which was preliminarily diagnosed as a thymic tumor before surgery and thymic squamous carcinoma after surgery. This study aimed to investigate the suitability of a modified subxiphoid enlarged thymectomy for thymic carcinoma and the pathological features of squamous thymic carcinoma. In addition, the evidence-based medical literature related to thymic tumor surgery was also reviewed.

## Methods

**Fig. 1:**
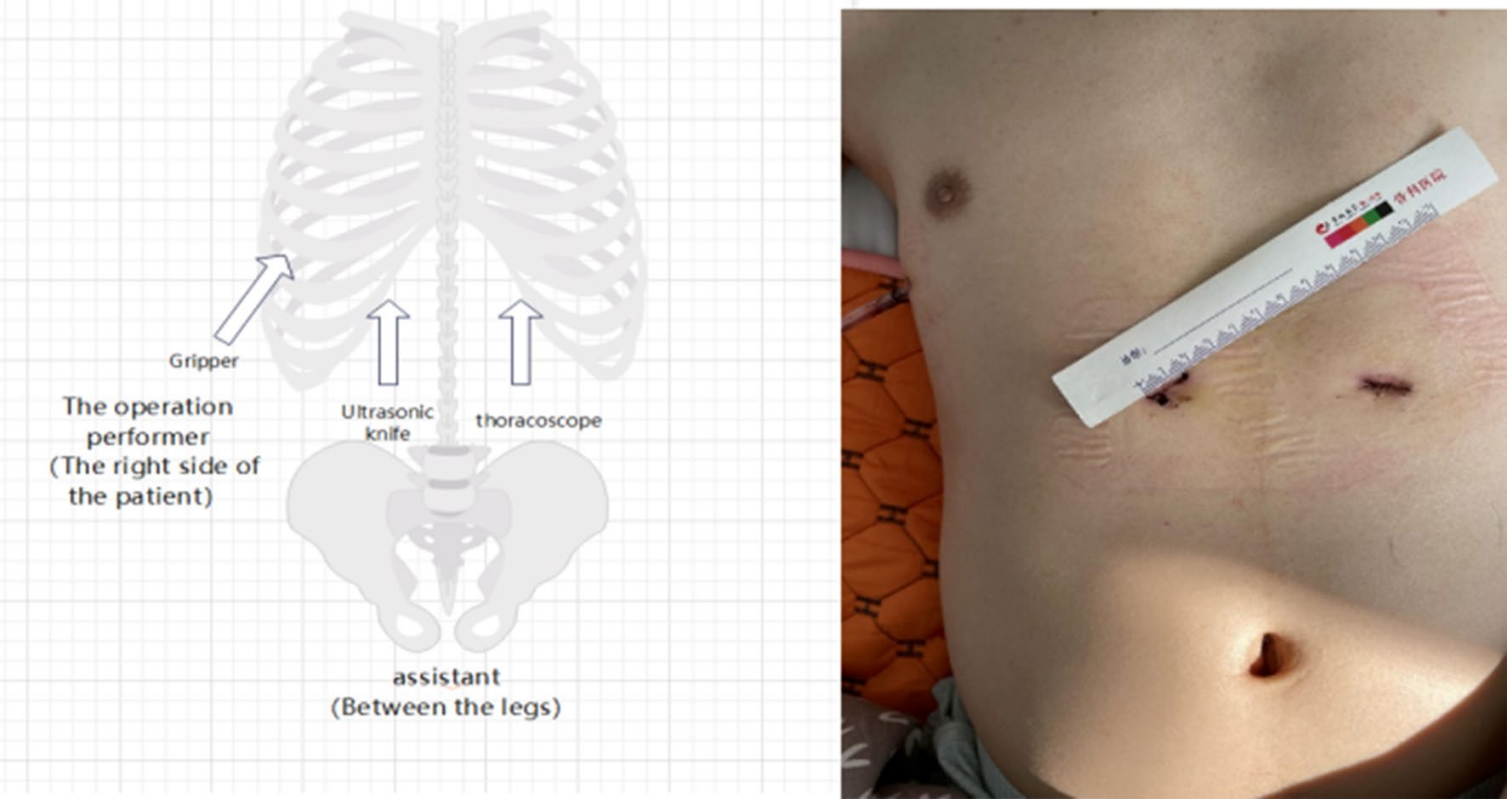
The position of the three incisions and the standing position of the surgeon and his assistant

The patient was placed in a supine position on the operating table with the legs in a split-leg position, the operator on the patient's right side, and the assistant in the split-leg position. Ventilation of both lungs was provided by a single-lumen endotracheal tube under general anesthesia. The resected region included the upper pole of the lower pole of the thymus and the thymic tumor, which included the mediastinal pleura and the diaphragmatic fatty tissue of the ribs. A 1.5-cm incision was made at each of the bilateral costal arches 3 cm from the glabella (Fig. 1).

The rectus abdominis muscle was dissected, the posterior sternal space was created by finger dissection, and separate poke cards were placed. A thoracoscope was disposed of at the left costal arch incision, and 8–12 cm H_2_O positive pressure carbon dioxide (CO_2_) was injected to create mediastinal emphysema, enlarging the posterior sternal space and facilitating thymic dissection. An ultrasonic knife was disposed of in the right costal arch incision to separate the mediastinal pleura bilaterally. A 0.5-cm incision was made at the junction of the right 6th rib and the anterior axillary line, and a poke card and grasping forceps were placed. The lower pole of the thymus was first lifted with the grasping forceps to reveal the ascending aorta and the innominate vein. At the same time, the fat pad near the diaphragmatic nerve was dissected with an ultrasonic knife. The anterior pericardial fat and the fat pad near the diaphragmatic angle of the heart, the pulmonary window of the aorta, and the lower extremity of the thyroid were carefully dissected. All specimens were placed in the specimen bag, the catheter was carefully placed in the right rib arch incision, and the thoracoscope was removed. Skeletonization of the superior vena cava, innominate vein, and aorta was revealed. The drainage tube was inserted diagonally upward into the mediastinum through an incision at the junction of the right 6th rib and axillary front, and the skin incision was sutured [6].

Patients and families were made aware of the advantages and risks of this new approach before surgery, as with the possibility of being switched to a sternal approach if a larger blood vessel was injured or if a new procedural approach was not an option, and informed consent was obtained. In addition, written informed consent was obtained from each patient, and ethical approval was obtained from the Research Ethics Committee of the Second Hospital of Jilin University.

## Case report

**Fig. 2:**
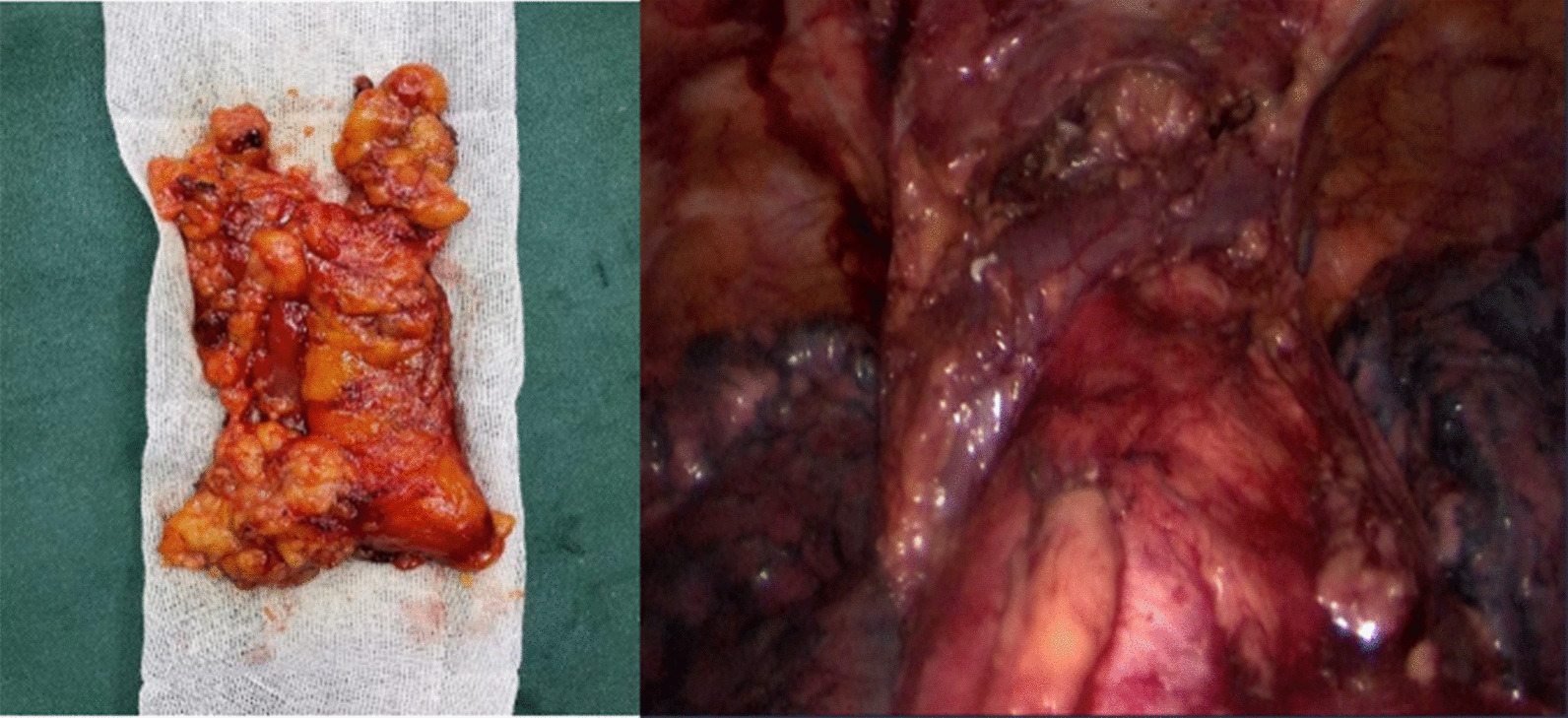
Left: Thymic carcinoma; Right: Left innominate vein, pericardium, and inferior pole of thyroid were exposed

A 63-year-old man was admitted to the Department of Thoracic Surgery of the Second Hospital of Jilin University (Jilin, China) 2 months after a physical examination for a mediastinal mass. All relevant examinations were completed after admission at the hospital. A chest computed tomography (CT) scan revealed a soft tissue shadow of a round shape of about 17 mm × 24 mm. Multiple nodules could be seen in both lungs; the larger ones were about 4 mm in diameter. Systemic bone imaging and abdominal CT scan showed no obvious metastatic lesions. Only cytokeratin 19 fragments of tumor markers were slightly above the reference range. After completing all investigations, a modified subxiphoid subcostal thymectomy was performed under general anesthesia with a single-lumen tracheal tube. A solid mass of 3.0 cm × 2.0 cm × 3.0 cm was removed and sent for pathological examination (Fig. 2).

**Fig. 3:**
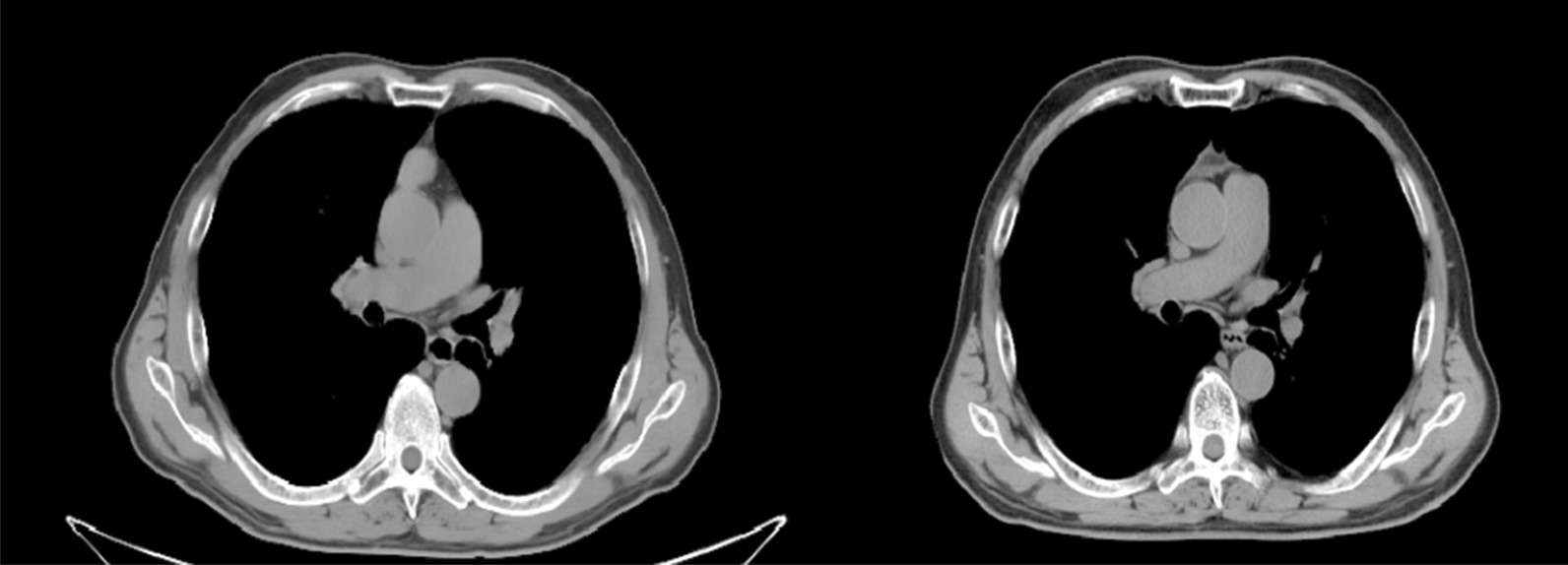
Left: In the anterior mediastinum, there was a soft tissue shadow of round shape, with a size of about 17 mm × 24 mm, and a computed tomography (CT) value of about 62 HU Right: One year after surgery, the chest CT showed the density of patchy fluid in the anterior mediastinum.

Postoperative pathological report: This (moderately differentiated) squamous cell carcinoma infiltrates the surrounding adipose tissue but does not infiltrate blood vessels. The patient was diagnosed with thymus carcinoma (Masaoka-Koga, stage II_b_) [7]. Immunohistochemical staining results: CK (AE1/AE3) ( +), CD117 ( +), CD1a (in +), Ki67 (positive rate 10%), P40 ( +), about the ( +), CK5/6 ( +), the TdT (−), EMA (−), EBER (−), CD5 ( +), CD99 (−). A bedside chest radiograph on postoperative day 1 showed good bilateral lung distention and sharp bilateral costophrenic angles. The thoracic drainage tube was routinely removed on the second day after surgery. He then received radiation and five cycles of chemotherapy, albumin combined with paclitaxel (400 mg every 4 weeks on day 1) and nedaplatin (110 mg every 4 weeks on day 2). One year after surgery, the patient was in good condition without complaints of discomfort. Chest CT revealed a patchy fluid density in the anterior mediastinum (Fig. 3).

## Discussion

Thymic carcinoma is a rare, highly malignant solid tumor originating in the thymic epithelium with malignant cytological characteristics and a high potential for invasion and metastasis. Thymus carcinoma still ranks among the top 10 thoracic neoplasms in terms of mortality. Thymic squamous cell carcinoma (TSCC) is the predominant pathological category of thymic carcinoma, representing an estimated 80% of all thymic carcinomas [2, 8]. Compared to other thymic carcinomas, TSCC exhibited less aggressive behavior with a 5% reduction in OS rates at 5 years (59.5%) and 8 years (54.5%) [9]. CD5 and CD117 are generally used as diagnostic markers for TSCC; however, lung squamous cell carcinoma (LSCC) occasionally expresses CD5 (0–15%) or CD117 (6.2–20%) [10, 11]. In contrast, one case of CD70-positive and CD5/ CD117-negative was observed by Jumpei Kashima et al. These results suggest that each marker cannot completely distinguish TSCC from LSCC independently [12]. Meanwhile, Yohei Taniguchi et al. showed that preferentially expressed antigens in melanoma (PRAME) may be a new TSCC-specific diagnostic marker. This study showed that of the 17 cases of TSCC and 116 thymomas in which PRAME expression was detected by immunohistochemistry, all 17 cases (100%) of TSCC showed diffuse strong expression of PRAME. In comparison, eight cases (6.8%) of the 116 thymomas showed weak focal expression. Seventeen cases (100%) and 16 cases (94.1%) of TSCC were CD117 and CD5 positive, and 1 (0.9%) case of B3 thymoma was double positive for CD5 and CD117. CD5/CD117 positive B3 thymoma was PRAME negative [13]. Consequently, the evaluation of CD70 in combination with CD5 and CD117 or PRAME in combination with CD5 and CD117 may help to diagnose TSCC more accurately. Notably, the independent prognostic factors associated with overall survival were age at diagnosis and SEER stage [14].

However, due to the rarity of thymic carcinoma, a standard postoperative adjuvant protocol remains to be established. One study confirmed that postoperative radiotherapy reduces recurrence of thymic malignancies [15]. In the latest version of the National Comprehensive Cancer Network guidelines, the paclitaxel + carboplatin regimen is recommended as the first-line chemotherapy regimen for TC. This recommendation is based on a phase II study that reported an ORR of 21.7% and a median PFS of 5.0 months with paclitaxel combined with platinum in patients with advanced TC [16]. A subgroup analysis of a phase III study in patients with NSCLC showed a significantly better response to platinum in combination with nab-PAC in squamous cell carcinoma [17]. In this case, adjuvant radiotherapy and chemotherapy were given to the patient, but more clinical trials are needed to verify the effectiveness and safety of this modality.

Based on various genetic and histological data, Chun Jin et al. found that multiple thymic tumors may come from the same tumor clone, suggesting that some thymic tumors in multiple TSCC may be the metastases of the primary tumor. These findings contribute to our understanding of thymic epithelial neoplasms. Therefore, the potential association between primary tumor and metastasis reminds us that there may be communication channels in the thymus lobules that promote tumor recurrence and metastasis. This communication channel may exist in TSCC and thymoma. The findings suggest that, whether thymic carcinoma or thymoma, thymectomy is necessary to reduce the possibility of tumor recurrence and improve survival [18]. In addition, expanded thymectomy can be the most acceptable and effective treatment for thymic carcinoma [19].

Some studies have suggested that histological classification, complete resection, large vessel invasion, or adjuvant therapy impacted the prognosis of thymic cancer. However, few reports have been used for subtype analysis because of the low incidence rate. Patients receiving treatment should achieve local and complete control of tumors and maintain physiological function and quality of life. The treatment model, including surgery, has a considerable impact on the survival of patients. In many studies, total resection is recommended without complex diffusion and metastasis at diagnosis, which is considered a prognostic factor in patients with thymic cancer [20]. In this operation, the preoperative and intraoperative judgment was thymoma, so routine lymph node evaluation was not performed for the time being. Meanwhile, lymph node metastasis is an important factor affecting the prognosis of thymic malignancies. Although lymph node dissection does not improve the long-term prognosis of thymic malignancies, lymph node dissection plays a role in accurate staging and improved prognostic prediction [21]. Therefore, it is necessary to perform expanded thymectomy and lymph node dissection during surgery.

Although thymic carcinoma is considered a highly aggressive tumor, surgical treatment combined with chemotherapy and radiation provides good long-term results for thymus cancer [4]. Therefore, the primary treatment of choice for thymic carcinoma is complete excision. Thymic carcinoma has a postoperative 5-year survival rate of 58–80%, and postoperative complications and mortality are rarely reported [8, 9, 22, 23].

Various approaches for thoracoscopic resection of thymic tumors have been developed, including median sternotomy, unilateral, bilateral, subxiphoid single-port, and triple-incision approaches [6, 24–28].

Whether it is thymoma or thymic carcinoma, median sternotomy is the preferred surgical method, but it has disadvantages such as large postoperative trauma, heavy postoperative pain, and an unaesthetic scar [29]. Therefore, minimally invasive surgical techniques for thymus gland disease treatment are very important. Right thoracoscopic thymectomy with less invasive approach is now being accepted by more and more thoracic surgeons [30, 31], but the left side of the thymus cannot be completely removed. Thus, to date, there is no consensus on the standard thoracoscopic approach for thoracoscopic thymectomy, especially for enlarged thymectomy. Surgeons usually choose a thoracoscopic approach based on their training experience and preferences. Marcin Zieliński et al. [32, 33] used double raising of the sternum in thoracoscopic thymectomy, which required the combination of transcervical and subxiphoid incisions. Learning was complicated, requiring too much time and too many incisions.

Marcin Zieliński et al. [25] used a sternal-assisted retractor with incisions above the sternal notch and below the xiphoid process to better expose the anterior mediastinum. Still, its disadvantages were that there were too many incisions, a large trauma surface, and poor postoperative appearance. Ren Xiang Jia et al. [34] created a 3-mm port at the level of the right third intercostal paraspinal line to elevate the sternum based on thoracoscopic thymectomy of the right thorax, and the fatty tissue of the upper pole of the thymus and the left costal diaphragm could be seen, but it is difficult to completely expose the contralateral side and remove all mediastinal adipose tissues. Francesco Caronia et al. [35, 36] studied bilateral thoracoscopic thymectomy. Although bilateral thymus tissues and fat pads can be more effectively removed compared with the above-mentioned procedure, more incisions are required, which may increase surgical trauma and postoperative pain. Qiang Lu et al. [6] investigated the treatment of myasthenia gravis with a “three-port” thoracoscopic expanded thymectomy under the costal arch under the xiphoid process, in which carbon dioxide was injected into the thoracic cavity to elevate the sternum and expose the anterior mediastinal space. Its advantages lie in the excellent visualization of the entire anterior mediastinum, facilitating removal of fatty tissues and minimizing the chance of accidental surgical injuries, such as accidental blood vessel tear or bilateral diaphragmatic nerve injury. Furthermore, single-lumen double-lung ventilation can be applied to patients who cannot tolerate single-lung ventilation and have a poor cardiopulmonary function, increasing the chances of surgery for more patients. However, its disadvantage lies in the relatively close location of the three incisions under the costal arch under the xiphoid process, which greatly affects the coordination of surgical instruments and the thoracic lens.

Therefore, based on the premise of the three-incision surgery, we proposed a new, improved thoracoscopic extended thymectomy under the xiphoid subcostal arch, which removed the 3-cm incision under the xiphoid process, retained the 1-cm incision of the bilateral costal arch, and added a 5-mm incision at the right 6th intercostal junction with the axillary front. Compared to the ideas proposed by previous investigators, the new approach ensures an adequate exposure of the anterior mediastinum, bilateral phrenic nerves, left innominate vein, right internal mammary vein, and bilateral pericardial fat pads, allows more patients who are unable to tolerate single-lung ventilation to have access to the procedure, and facilitates the operator's manipulation without interfering with the assistant's instruments as much as possible. By changing the thoracoscopic entrance to the bilateral costal arches and retraction, the supra-diaphragmatic adipose tissue and thymic tissue around the main-pulmonary artery window can be better exposed for the purpose of enlarged thymectomy. In addition, a chest drain is placed diagonally upwards through the 6th intercostal space to allow for better drainage of gas and fluid, reducing the length of stay and improving patient satisfaction. Therefore, the modified subxiphoid subcostal arch thoracoscopic enlarged thymectomy is not only suitable for TSCC but also for thymic cysts, thymomas, locally invasive thymomas, and thymic carcinomas, thus providing new options to surgeons. However, large-scale randomized and controlled clinical trials are necessary to demonstrate the procedure's safety and efficacy, and this surgical method still has some shortcomings. First, the diameter of the thymic tumor is too large to be removed from the minimally invasive incision. Second, subxiphoid surgery is not suitable for patients with large hearts who do not fully expose the anterior mediastinum. Third, midline sternotomy is still recommended for severe intraoperative thymic carcinoma involving the heart and large vessels.

## Conclusion

The evaluation of CD70 in combination with CD5 and CD117 or PRAME in combination with CD5 and CD117 may help to diagnose TSCC more accurately. In addition, the modified thoracoscopic expanded thymic resection under the costal arch of the xiphoid process is not only suitable for TSCC but also for thymic cysts, thymomas, and locally invasive thymomas and thymic carcinomas, which provides new options for surgeons.

## Data Availability

Data sharing is not applicable to this article as no datasets were generated or analyzed during the current study.
